# PEGylated recombinant human hyaluronidase (PEGPH20) pre-treatment improves intra-tumour distribution and efficacy of paclitaxel in preclinical models

**DOI:** 10.1186/s13046-021-02070-x

**Published:** 2021-09-10

**Authors:** Lavinia Morosi, Marina Meroni, Paolo Ubezio, Ilaria Fuso Nerini, Lucia Minoli, Luca Porcu, Nicolò Panini, Marika Colombo, Barbara Blouw, David W. Kang, Enrico Davoli, Massimo Zucchetti, Maurizio D’Incalci, Roberta Frapolli

**Affiliations:** 1 Istituto di Ricerche Farmacologiche Mario Negri IRCCS, Department of Oncology, via M. Negri 2, 20156 Milan, Italy; 2 Present address: IRCCS Humanitas Research Hospital, Via Manzoni 56, 20089 Rozzano, Milan, Italy; 3 Department of Veterinary Medicine, University of Milan, Lodi, Italy; 4 Mouse and Animal Pathology Laboratory (MAPLab), Fondazione UniMi, Milan, Italy; 5 Biocept, San Diego, California USA; 6 Halozyme Therapeutics, San Diego, California USA; 7 Istituto di Ricerche Farmacologiche Mario Negri IRCCS, Laboratory of Mass Spectrometry, Milan, Italy; 8 Present address: Department of Biomedical Sciences, Humanitas University, Via Rita Levi Montalcini 4, 20090 Pieve Emanuele, Milan Italy

**Keywords:** Mass spectrometry imaging, Drug distribution, Extracellular matrix, Hyaluronan, Solid tumours

## Abstract

**Background:**

Scarce drug penetration in solid tumours is one of the possible causes of the limited efficacy of chemotherapy and is related to the altered tumour microenvironment. The abnormal tumour extracellular matrix (ECM) together with abnormal blood and lymphatic vessels, reactive stroma and inflammation all affect the uptake, distribution and efficacy of anticancer drugs.

**Methods:**

We investigated the effect of PEGylated recombinant human hyaluronidase PH20 (PEGPH20) pre-treatment in degrading hyaluronan (hyaluronic acid; HA), one of the main components of the ECM, to improve the delivery of antitumor drugs and increase their therapeutic efficacy. The antitumor activity of paclitaxel (PTX) in HA synthase 3-overexpressing and wild-type SKOV3 ovarian cancer model and in the BxPC3 pancreas xenograft tumour model, was evaluated by monitoring tumour growth with or without PEGPH20 pre-treatment. Pharmacokinetics and tumour penetration of PTX were assessed by HPLC and mass spectrometry imaging analysis in the same tumour models. Tumour tissue architecture and HA deposition were analysed by histochemistry.

**Results:**

Pre-treatment with PEGPH20 modified tumour tissue architecture and improved the antitumor activity of paclitaxel in the SKOV3/HAS3 tumour model, favouring its accumulation and more homogeneous intra-tumour distribution, as assessed by quantitative and qualitative analysis. PEGPH20 also reduced HA content influencing, though less markedly, PTX distribution and antitumor activity in the BxPC3 tumour model.

**Conclusion:**

Remodelling the stroma of HA-rich tumours by depletion of HA with PEGPH20 pre-treatment, is a potentially successful strategy to improve the intra-tumour distribution of anticancer drugs, increasing their therapeutic efficacy, without increasing toxicity.

**Supplementary Information:**

The online version contains supplementary material available at 10.1186/s13046-021-02070-x.

## Background

Many molecular mechanisms responsible for resistance to anticancer drugs have been elucidated over the years. The co-existence of heterogeneous populations of cancer cells with different sensitivity explains why, even after an initial response, most solid tumours relapse, becoming resistant to treatments.

Growing evidence suggests that the resistance of many solid tumours can also be due to insufficient and heterogeneous tumour drug distribution [1, 2]. Studies on preclinical and clinical tumours after treatment indicate that the loss of the normal tissue architecture hampers drug penetration in cancer tissues [3–13].

The penetration capacity of a drug depends on its physical-chemical properties and on the neoplastic cells’ characteristics, but the low delivery in tumour tissue is mainly related to the altered tumour microenvironment (TME) [2]. Abnormal blood and lymphatic vessels, the reactive stroma, and inflammation characterizing the neoplastic phenotype, causes an increase in solid stress, hypoxia and tumour interstitial fluid pressure (TIFP), primary obstacles to the delivery of therapeutics [5, 13]. Moreover, solid tumours often have a desmoplastic stroma composed of a dense fibrous connective matrix made of proteoglycans, hyaluronan (HA), fibrous proteins (e.g. collagen) and stromal cells [14]. This abnormal extracellular matrix (ECM) further increases TIFP and solid stress, causing frictional resistance to penetration [15–17]. These alterations typical of the TME affect the uptake, distribution and efficacy of anticancer drugs [18–20].

HA is a glycosaminoglycan and a major component of the normal ECM, whose accumulation is significantly increased in several solid malignancies, particularly in advanced stage disease [21]. High HA levels are associated with cancer aggressiveness and worse prognosis in pancreatic, breast, ovarian, prostate, gastric, colorectal and lung cancers [22, 23]. An increase in HA accumulation is caused by an imbalance between production and degradation by endogenous enzymes [24]. HA is a ligand of CD44 and RHAMM receptors, that mediates cell proliferation, invasion and inflammation, and constitutes a sort of glycocalyx on the cell surface, allowing cells to evade anoikis and death caused by external stimuli [21]. Its charge and ability to form high molecular weight aggregates with other ECM components, enable extracellular HA to trap water molecules, promoting an increase of TIFP [25]. It forms a barrier that restricts the delivery of antibodies and immune cells to tumours [26].

To improve the delivery of antitumor drugs and increase their therapeutic efficacy, some strategies based on ECM modification have been tested. Lowering HA levels in TME with a hyaluronidase has been suggested as a “distribution enhancing approach”. By cleaving polymeric HA into substituent units, hyaluronidase can liberate trapped water molecules, thus rapidly lowering TIFP and enabling collapsed vessels in the tumour to open. Thus, it would improve tumour perfusion and drug permeation. rHuPH20, a recombinant human hyaluronidase approved for subcutaneous injection to facilitate the absorption of other injected drugs (e.g. local anaesthetics) or fluids, has been further developed for anticancer purposes. PEGPH20 is an investigational PEGylated form of rHuPH20 that has a longer plasma half-life (about 10.3 h for PEGPH20 compared to 3 min for rHuPH20 in mice) and it is suitable for intravenous administration [27]. In preclinical studies, the pharmacodynamic activity of PEGPH20, in different tumour, showed enhanced tumour perfusion and therapeutic activity of co-administered cytotoxic drugs [15, 27, 28]. Additionally, PEGPH20 at a high dose (1 mg/kg) increased the entry of therapeutic antibodies, immune cells and small molecules into the tumour stroma [25, 26, 29, 30].

In patients, PEGPH20 is tolerated, although prophylactic steroids and anticoagulant are required to prevent musculoskeletal and thromboembolic events [31, 32]. Tumour HA levels have been proposed as a predictive marker, since major PEGPH20 antitumor activity was observed against cancers expressing high HA levels [33]. Nevertheless, the HALO 109–301 trial indicated that PEGPH20 plus standard chemotherapy in patients with HA-high metastatic pancreatic ductal adenocarcinoma (PDA) increased overall response but not overall survival and progression free survival [34, 35], suggesting that for this tumour the increase in drug concentration is not sufficient to overcome the resistance to therapy. The lack of effect of PEGPH20 in pancreatic cancer does not justify stopping its development for other tumours that are not as refractory to chemotherapy as PDA and in which the higher drug concentration could effectively improve the antitumor activity.

A fuller description of the connections between drug distribution, TME characteristics and antitumor activity could help elucidate the true potential of this therapeutic approach. We investigated the distribution of paclitaxel (PTX) after PEGPH20 pre-treatment using mass spectrometry imaging (MSI) and correlated it with antitumor activity and tissue modifications in different preclinical models.

## Methods

### Drugs and reagents

Paclitaxel (PTX, Indena S.p.A., Milan, Italy) and paclitaxel-D5 (D5-PTX, Alsachim, France) were dissolved in ethanol at a concentration of 1 mg/ml. Serial dilutions of drug were prepared in 50% ethanol from 0.5 to 100 pmol/μl for all MSI experiments.

For treatment purposes, PTX was dissolved in 50% Cremophor EL (Sigma) and 50% ethanol and further diluted 1:5 in saline immediately before use.

The PEGPH20 formulation (provided by Halozyme Therapeutics Inc.) consisted of the active compound dissolved in histidine buffer (histidine 10 mM, NaCl 130 mM, pH 6.5) at 5 mg/ml. It was diluted in saline solution (NaCl 154 mM) just before the treatment.

TiO_2_ nanoparticles (Aeroxide TiO_2_ P25, Evonik Industrials, Essen, Germany) were used as a matrix for MSI experiments, dissolved at the concentration of 1 mg/ml in ethanol 50%/KCl 0.5%. The TiO_2_ nanoparticle suspension was vortexed and sonicated for 3 min just before use, to reduce agglomeration and sedimentation.

### Cell lines

Parental SKOV3 (SKOV3) and HAS3-overexpressing SKOV3 (SKOV3/HAS3) cell lines were provided by Halozyme Therapeutics Inc. SKOV3/HAS3 were obtained by transducing SKOV3 cells with a retrovirus carrying human HA synthase 3 (HAS3) cDNA. These cells are characterized by production of high HA levels. Both cell lines were maintained in McCoy medium added with 10% FBS and glutamine. The BxPC3 pancreatic adenocarcinoma cell line was maintained in RPMI with 10% FBS and glutamine. All the cell lines were mycoplasma free and authenticated by short-tandem repeat (STR) profiling.

### Animals

Six- to 7-week-old female NCr*-nu/nu* mice were obtained from Envigo RMS. They were maintained under specific pathogen-free conditions, housed in isolated vented cages, and handled using aseptic procedures after an acclimatization period of one week.

All procedures involving animals and their care were conducted in conformity with the following laws, regulations, and policies governing the care and use of laboratory animals: Italian Governing Law (D.lgs 26/2014; Authorization n.19/2008-A issued March 6, 2008 by Ministry of Health); Mario Negri Institutional Regulations and Policies providing internal authorization for persons conducting animal experiments (Quality Management System Certificate – UNI EN ISO 9001:2008 – Reg. N° 8576-A); the NIH Guide for the Care and Use of Laboratory Animals (2011 edition) and EU directives and guidelines (EEC Council Directive 2010/63/UE) and in line with Guidelines for the welfare and use of animals in cancer research {Workman, 2010}. Animal experiments were reviewed and approved by the IRFMN Animal Care and Use Committee (IACUC) that included “ad hoc members for ethical issues. Animals were housed in the Institute’s Animal Care Facilities, which meet international standards; they are regularly checked by a certified veterinarian who is responsible for health monitoring, animal welfare supervision, and experimental protocols and procedures revision. Experiments were reported following the *ARRIVE guidelines 2.0* (Animal Research: Reporting of In Vivo Experiments).

### Antitumor activity

Tumour cells (5 × 10^6^ cells) were implanted subcutaneously in the flank of nude mice. Tumour growth was measured with a Vernier caliper two/three times a week, and tumour weights (1mm^3^ = 1 mg) were calculated as [length (mm) * width^2^ (mm^2^)]/2. When tumours reached approximately 150–200 mg, mice were assigned to the experimental group by stratified randomization based on tumour weight (8 to 9 mice/group) and treated with PEGPH20 (0.1 mg/kg, intravenous bolus, once a week for two doses) alone or with PTX (20 mg/kg, intravenous bolus PEGPH20, once a week for two doses) or with PEGPH20 followed by PTX (20 mg/kg, intravenous bolus, 24 h after each dose of 0.1 mg/kg PEGPH20, once a week for two doses). Control groups received the same volume of the respective vehicles. Mice were weighted two/three times a week to evaluate general toxicity. Tumour volume was measured thrice a week up to the ethical endpoint (1500 mm^3^) then mice were euthanized. For the analysis of the tumour growth curves, each tumour weight (TW) measure was normalized to the tumour weight of the same mouse at the start of treatment (relative tumour weight; RTW). Antitumor efficacy was expressed as best T/C% were T and C were the mean of the RTWs of treated and control mice, respectively according to the standards of the National Cancer Institute (NCI) of the United States [36]. For both treatments and measurements, mice were randomly collected from their cage. More detailed analysis of the tumour growth curves is given in Additional file 1.

### Mass spectrometry imaging

Mice bearing SKOV3 or SKOV3/HAS3 tumours weighting approximately 200–500 mg were treated with PTX (60 mg/kg, single dose or 20 mg/kg followed by a second dose of 60 mg/kg a week later), alone or after PEGPH20 (0.1 mg/kg, 24 h before each dose of PTX) (3 or 4 mice/group). Mice were euthanized 4 h after the last dose of PTX and plasma was collected as described for pharmacokinetic analysis. Tumours were explanted and divided into two parts: the first was immediately snap-frozen in liquid nitrogen and stored at − 80 °C until MSI analysis, and the second was stored at − 20 °C for HPLC analysis.

For MSI analysis of PTX distribution, a method developed in our laboratory was used [37, 38]. Briefly, frozen tissues were cut in 10 μm thick sections using a cryomicrotome (Leica Microsystems, Wetzler, Germany) at − 20 °C. One section, every 300 μm apart, was cut starting from the central part of the tumour. Each section was mounted on a pre-cooled MALDI plate (Opti-TOF 384 Well insert) by standard thaw-mounting techniques. The two adjacent sections were mounted on a glass slide for histopathological analysis (as described below) and stored at − 20 °C. 3–4 tumours/group and 3 sections/tumour were analyzed. The MALDI plate was dried under vacuum at room temperature for 1 h, then sprayed with TiO_2_ matrix suspension with deuterated PTX (D5-PTX, 3 μg/mL), as internal standard. A BD 180 precision double-action trigger airbrush with a 0.20 mm nozzle diameter, and nitrogen at 0.2 atm was used. A MALDI 4800 TOF-TOF (AB SCIEX Old Connecticut Path, Framingham, MA 01701, USA) was used, equipped with a 355 nm Nd:YAG laser with a 200 Hz repetition rate, controlled by the 4000 Series ExplorerTM software (AB SCIEX Old Connecticut Path, Framingham, MA 01701, USA). Images of tissue sections were acquired using the 4800 Imaging Tool software (www.maldi-msi.org, M. Stoeckli, Novartis Pharma, Basel, Switzerland), with an imaging raster of 100 × 100 μm.

A custom pre-processing pipeline [39] was used to analyse the MSI data and generate the drug distribution images. The distribution of the PTX ion signal in the tumour image, outlined by drawing a ROI based on a tissue-associated signal (m/z = 281.27), was analysed calculating the mean and CV% of the drug concentration at pixel level and grey-level size-zone matrix (GLSZM) features. A GLSZM quantifies grey level zones in an image. A grey level zone is defined as series of connected pixels that share the same grey level intensity, making GLSZM rotation-independent [40]. A panel of GLSZM-derived features (including the recently proposed drug homogeneity index -DHI [41]) related to the size of the zones and grey-level intensity values was used to evaluate the tumour drug spatial distribution in the image. The mathematical formulas to calculate these features and their meaning are reported in Table 1.

**Table 1:** Grey-level size-zone matrix features

Abbreviation	Name	Description	Mathematical formula
ZP	Zone percentage	lower values indicate that the image is made of a few large zones with the same grey-level. Higher values indicate greater fragmentation of the image into small zones	nzones/npix
LZE	Large zone emphasis	associated with the presence of wide areas with similar drug concentrations, regardless of whether they are low or high	∑_ij_ Z_ij_j^2^ /nzones
HGZE	High Grey-level Zone Emphasis	indicative of the presence of areas with high drug concentrations, regardless of their size	∑_ij_ Z_ij_i^2^ /nzones
LZHGE	Large-zone High Grey-level Emphasis	focuses on the presence of wide areas with high drug concentrations	∑_ij_ Z_ij_j^2^ i^2^ /nzone
IV	Intensity variability	highest when there are few large zones with low drug concentrations. It decreases when the concentration increases and smaller zones are formed with higher drug concentrations	∑_i_ (∑_j_ Z_ij_)^2^/i^4^/nzones
GLNn	Grey-Level Non-uniformity normalised	highest when zones concentrate to a single grey level, lower when all grey levels are equally represented (poorly sensitive to redistribution among grey levels)	(∑_i_ (∑_j_ Z_ij_)^2^ /nzones)/nzones
ZSμ	Zone Size mean	average size of the zones, independently of the grey level. Strongly affected by the presence of a large number of small zones	∑_ij_ Z_ij_j /nzones
DHI	Drug-homogeneity index	a recently proposed feature measuring the average area of the larger zones (over a given arbitrary threshold ν) as a fraction of the ROI area	∑_i,j ≥ ν_ j Z_ij_ /∑_i,j ≥ ν_ Z_ij_ /npix

i: grey levels; j: zone sizes; nzones: total number of zones; npix: total number of pixels in the ROI

The total concentration of PTX in plasma and in the second part of the tumour sample was determined by HPLC as reported in our previous publications [42, 43].

### Histopathological analysis

Tumour slices adjacent to MSI slices were examined histopathologically. They were fixed in 10% neutral buffered formalin and stained with Hematoxylin-Eosin (H&E) or Alcian Blue pH 2.5 using standard techniques, and analysed under a light microscope. Analyses were blinded.

HA staining was performed using a Halozyme developed staining method [27]. Formalin-fixed paraffin embedded (FFPE) tumour sections were stained for HA using the immuno-adhesin HTI-601 with DAB used as the chromogen. Two slides were stained for every tissue sample including control slides. From each pair, one slide was pre-treated with recombinant human hyaluronidase PH20 (rHuPH20) in PIPES buffer at pH 5.5 to digest HA to create a negative control demonstrating the specificity of HTI-601 for each sample. The other slide from each pair was pre-treated with PIPES buffer alone, leaving the HA intact. A slide containing a liver section (HA negative) and a section from a BxPC3 xenograft (HA positive) were included as assay staining run controls. The staining run was considered acceptable when (a) the rHuPH20 pre-treated BxPC3 control section lacked HA staining, (b) the PIPES buffer pre-treated BxPC3 control presented HA staining, and (c) the liver section failed to show HA staining in hepatocytes. Stained slides were scored digitally for accumulation of HA using an algorithm from Aperio, Positive Pixel Count V9.

### Statistical analysis

The treatment effect on xenograft tumour growth curves was formally tested using a non-parametric approach. For each mouse, the partial tumour growth rates (k) in every interval [t_i_- t_i + 1_] were calculated as follows: k = [log TW_t(i + 1)_-log TW_t_]/ (t_(__i + 1)_-t_i_). The experimental groups were compared two by two using a Wilcoxon Rank-Sum test, stratified by intervals, on the obtained k values (SAS software, version 9.4). Student’s t-test for unpaired samples was used to evaluate differences in drug distribution experiments. Differences in the pharmacokinetic profiles were analysed by two-way ANOVA (Graph Pad Prism 8).

## Results

### PEGPH20 increases PTX antitumor activity in HAS3-overexperessing tumour

**Fig. 1:**
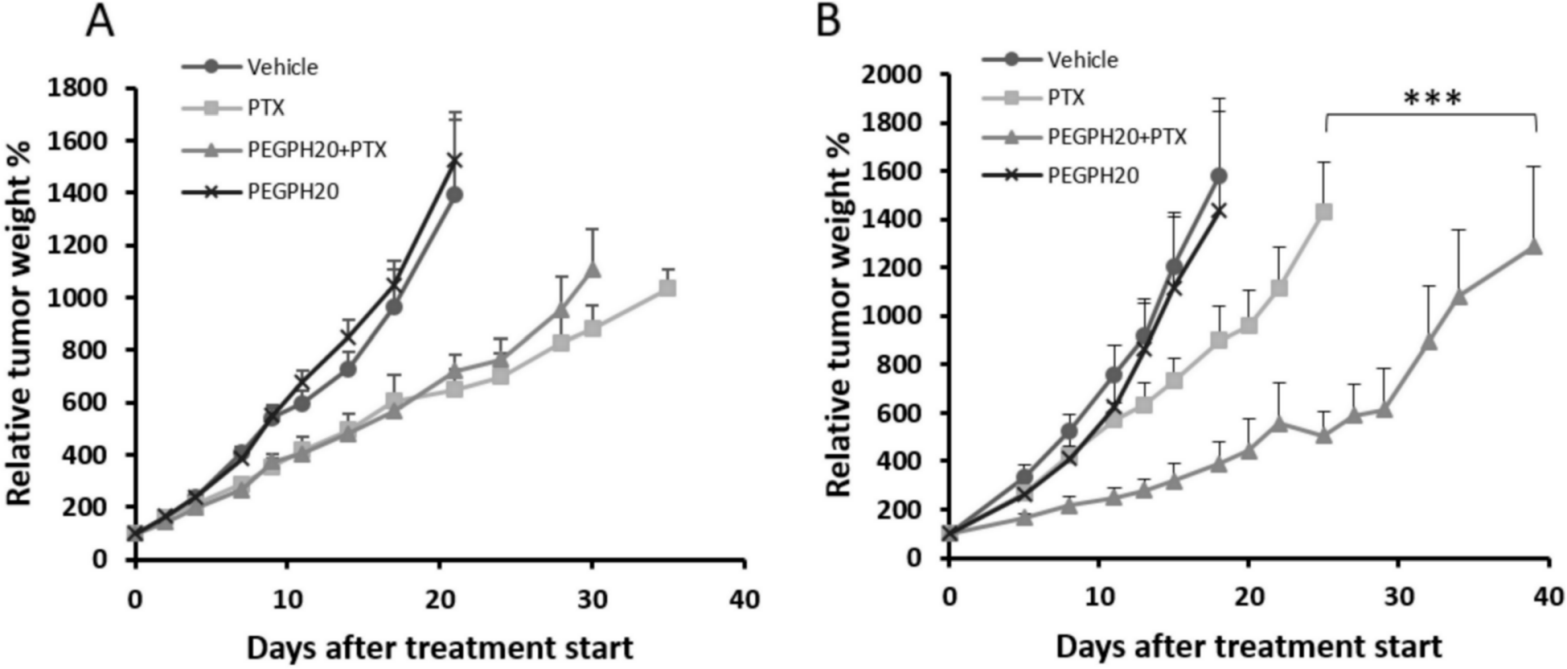
Antitumor activity of PEGPH20 and PTX in SKOV3 (**A**) and SKOV3/HAS3 (**B**) models. Tumour bearing mice (*n* = 9 and *n* = 8 in SKOV3 and SKOV3/HAS3 experiments, respectively) were randomized to receive PTX 20 mg/kg q7dx3 with or without the pre-treatment with PEGPH20 0.1 mg/kg, or PEGPH20 alone. In the SKOV3/HAS3 but not in the parental SKOV3 model, hyaluronidase combined with PTX dramatically enhanced the antitumor activity (** Wilcoxon Rank-Sum test stratified by intervals: *p*-value< 0.001 comparing the entire experimental groups)

The effect of PEGPH20 pre-treatment on PTX antitumor activity was assessed in parental and HAS3-overexpressing SKOV3 tumours (Fig. 1). PTX induced a modest reduction of tumour growth in both tumour models. However, this reduction is more evident in the parental cell line (best T/C% 46.6 on day 21 after treatment start and 56.9% on day 18 in SKOV3 and SKOV3/HAS3, respectively) consistently with a worse drug distribution in high HA tumours. The relevance of this data is further strengthened by results of an in vitro cytotoxicity study. Indeed, we found that the SKOV3-HAS3 were intrinsically more sensitive to PTX than the parental line, being the IC50 of PTX 200 nM and 300–400 nM in SKOV3-HAS3 and SKOV3 cell lines, respectively (Additional file 1). In SKOV3, PEGPH20 alone was completely inactive (best T/C 93.6% on day 7) and when given in combination did not affect the tumour growth inhibition induced by PTX (best T/C% 51.8 on day 21). Instead, in SKOV3-HAS3, although hyaluronidase was still inactive (best T/C% 78.2 on day 8) when combined with PTX it dramatically enhanced the antitumor activity (best T/C% 24.6 on day 18, *p*-value< 0.001 compared to control, p-value< 0.001 compared to single treatments). An in depth analysis of the tumour growth curves is shown in Additional file 1. Treatments were well tolerated, and no weight loss was observed.

### PEGPH20 effect on PTX distribution

**Fig. 2:**
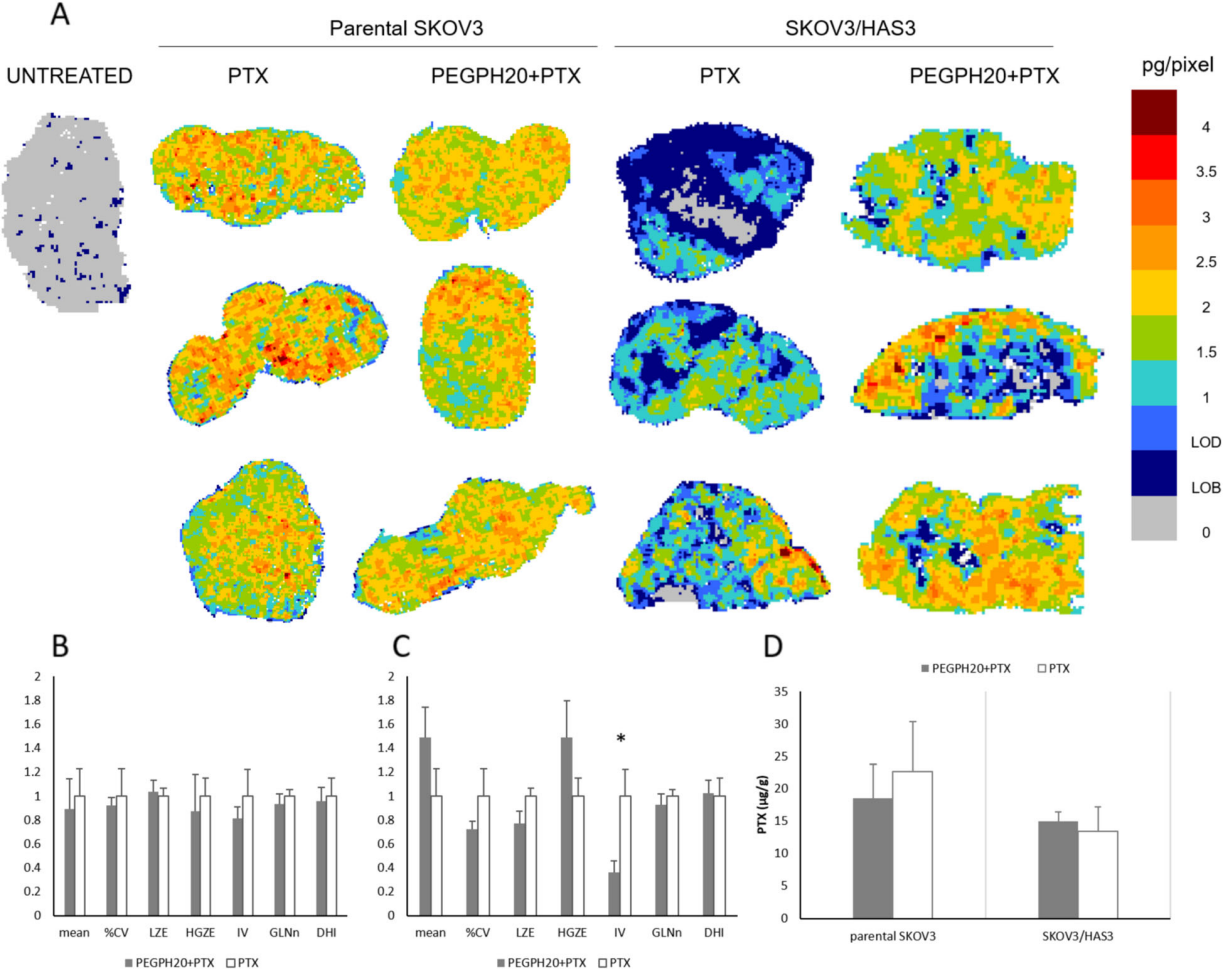
PTX distribution in SKOV3 and SKOV3/HAS3 tumors 4 h after a single PTX treatment, with or without PEGPH20 pre-treatment. Three tumours were analysed for each group. **A** Mass spectrometry images. One representative section of three analysed for each tumour is shown. **B** GLSZM features in SKOV3 and **C** SKOV3/HAS3 tumours. A selected panel of features (mean, CV%, LZE-Large-Zone Emphasis, HGZE-High Grey-level Zone Emphasis, IV-Intensity Variability, GLNn-normalised Grey-Level Non-uniformity and DHI-Drug Homogeneity Index) describing drug distribution and influenced by PEGPH20 pre-treatment is presented. The mean value of each feature was rescaled to the PTX mean for comparison (*p-value< 0.05). **D** Tumour concentrations of PTX measured by HPLC in the second half of the same tumours analysed for MSI

To verify the hypothesis that PEGPH20 enhances the PTX antitumor efficacy by improving its distribution in tumour tissue, we performed an in-depth analysis of spatial distribution of PTX within tumour tissue, using an in-house developed MSI technique. Measures of drug concentrations in tumour homogenates and plasma pharmacokinetics are not fully indicative of drug distribution in tumour tissue. Since heterogeneous penetration of pharmacological compounds in neoplastic tissue is a known mechanism of drug resistance, the determination of drug intratumor penetration adds valuable information. The MSI analysis of PTX is shown in Fig. 2. PTX administered as single dose alone or in combination efficiently penetrated the SKOV3 tumours, displaying quite homogeneous intra-tumour drug distribution. In the SKOV3/HAS model instead, there was an improvement in the PTX distribution after PEGPH20 pre-treatment. The distribution appeared uneven in tumours treated with PTX alone, with some areas where PTX concentrations were below the limit of detection (0.6 pg/pixel). After HA degradation by PEGPH20, the drug was distributed somewhat more evenly in the tumour tissue (Fig. 2A).

MSI images were further processed to extract quantitative parameters describing drug distribution. In addition to the mean and CV% of the drug concentration calculated at pixel level, we examined a panel of GLSZM features selected for their significance in relation to the diffusion and homogeneity of the drug in the tissue. The complete panel of GLSZM features and an interpretation of their meaning in this context is reported in Additional file 1. Figure 2B-C illustrates the features that are influenced by PEGPH20 pre-treatment in parental SKOV3 or SKOV3/HAS3. In parental SKOV3, the values of the features are almost undistinguishable in the two treatment groups, consistently with visual inspection of the MSI results, while in SKOV3/HAS3 there was a tendency alteration after PEGPH20 pre-treatment in some of the features. In SKOV3/HAS3 after PEGPH20 pre-treatment, the drug concentration at the pixel level measured by MSI (“mean” feature) was higher, with a lower %CV, suggesting more uniform drug diffusion. After PEGPH20 pre-treatment there was a statistically significant reduction of intensity variability (IV), a lower “large zone emphasis” (LZE) and a higher “high grey-level zone emphasis” (HGZE). Overall, this GLSZM features alteration induced by PEGPH20 pre-treatment highlighted fragmentation of the wide areas of lower drug concentration typical of tumours treated with PTX alone and an increase of higher drug concentration regions consistent with more widespread diffusion of the drug. However, the total drug concentration measured by HPLC in the second half of the same tumours used for MSI was not affected by PEGPH20 pre-treatment (Fig. 2D).

The complete pharmacokinetic analysis after a single dose of PTX 20 mg/kg, alone or after PEGPH20 pre-treatment in both tumour models is reported in the Additional Information. PEGPH20 pre-treatment minimally affect PTX pharmacokinetic behaviour. Indeed, PTX concentrations in tumour samples collected at different time points were comparable in the two different experimental groups (with or without PEGPH20 pre-treatment). Similar levels were also found for plasma in the SKOV3 model while circulating PTX was lower after PEGPH20 than vehicle pre-treatment in SKOV3/HAS3 model but only 1 h after PTX. Similarly, PTX levels in liver were lower in the combination in both experimental models 1 h after PTX.

**Fig. 3:**
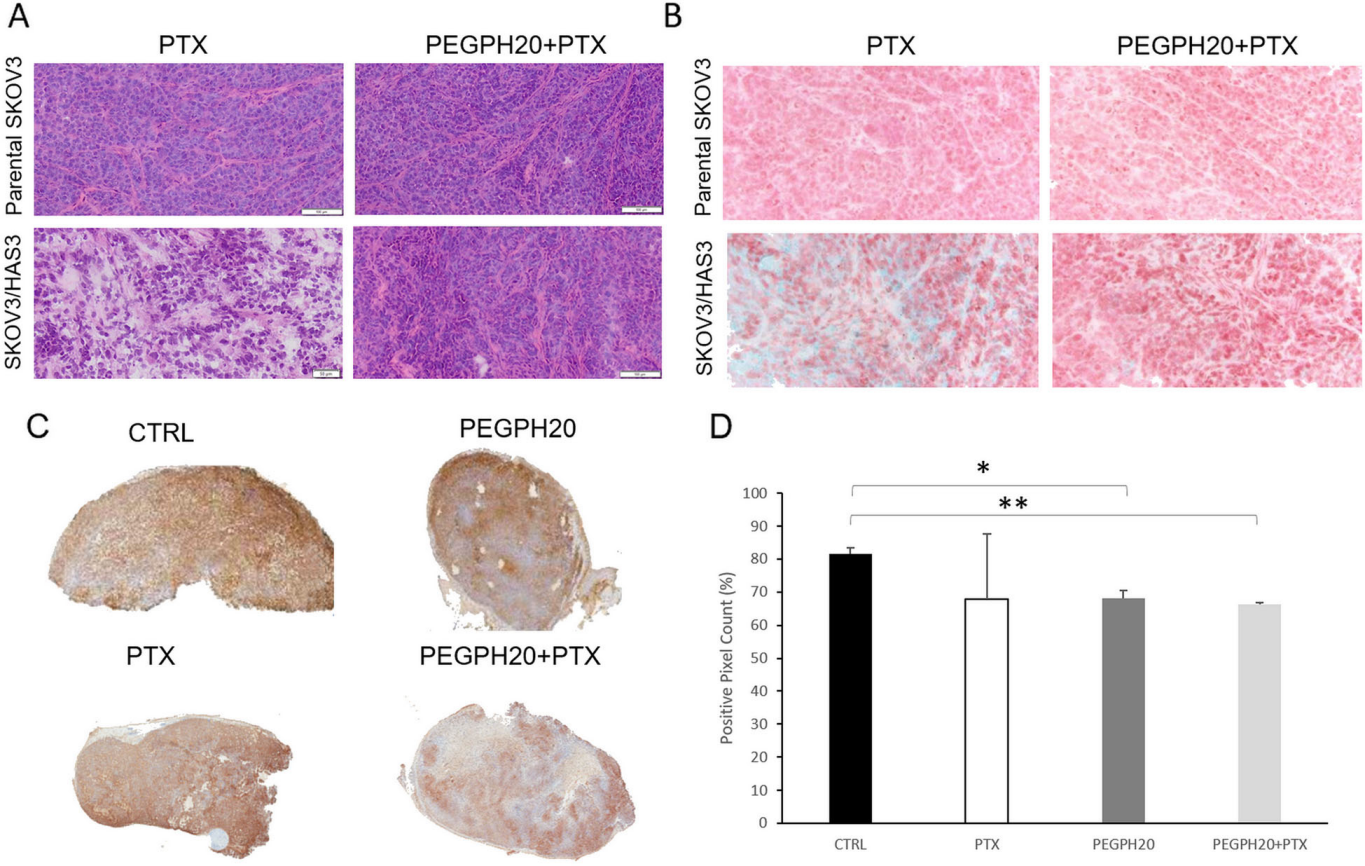
Representative images of the different tissue architecture of SKOV3 and SKOV3/HAS3 tumours after PEGPH20 and PTX treatment. **A** H&E staining, 100x; **B** Alcian Blue staining with nuclear red counterstain, 200x magnification; **C** representative HA staining images and **D** HA quantification in the different experimental groups (*Student's t-test: *p*-value< 0.05; **Student's t-test: *p*-value< 0.01)

To determine whether the different PTX distribution in tumour was due to differences in tissue architecture, we did histological examinations on tumour sections adjacent to those analysed by MSI. H&E staining confirmed that SKOV3 tumours were composed of sheets/solid areas of neoplastic cells arranged in lobules, infiltrated by a moderate to large amount of thin fibrovascular stroma and occasionally thicker fibrous septa (Fig. 3A and Additional file 1). Variably extensive areas of necrosis were also present. In this model, PEGPH20 and PTX did not induce any substantial morphological change. Similarly, no positivity for Alcian Blue was observed, staining acid mucosubstances and acetic mucins, with any treatment (Fig. 3B).

In SKOV3/HAS3 tumours, a looser arrangement of the tissue structure was evident, with loss of defined lobules. HA ability to bind large amounts of water molecules might be responsible for the looser arrangement of the tumour tissue observed in SKOV/HAS3 samples: a large amount of water is frozen and released during sample preparation, producing the artefacts characterized by clear/empty spaces dissecting the tumour tissue. The PEGPH20 pre-treatment restored a more compact tumour architecture, similar to that of parental SKOV3 tumours (Fig. 3A). No difference in the amount of necrosis and stroma was noted (Additional file 1). The widespread stromal component (recognized as Alcian Blue positivity) observed in SKOV3/HAS3 tumours was strongly reduced in PEGPH20-pretreated samples (Fig. 3B).

Specific HA depletion in SKOV3/HAS3 tumours following PEGPH20 treatment was quantified in tissue sections stained for HA using the immuno-adhesin HTI-601 with DAB used as the chromogen (Fig. 3C). A high HA content (expressed as positive pixel to HA presence, Fig. 3D) was found in untreated SKOV3/HAS3 tumours (81.6 ± 1.8%, mean ± sd) and a significant reduction in HA specific staining after PEGPH20 treatment (68.1 ± 2.5%, mean ± sd) could be detected (comparing untreated with PEGPH20 pre-treated samples, *p*-value = 0.025).

**Fig. 4:**
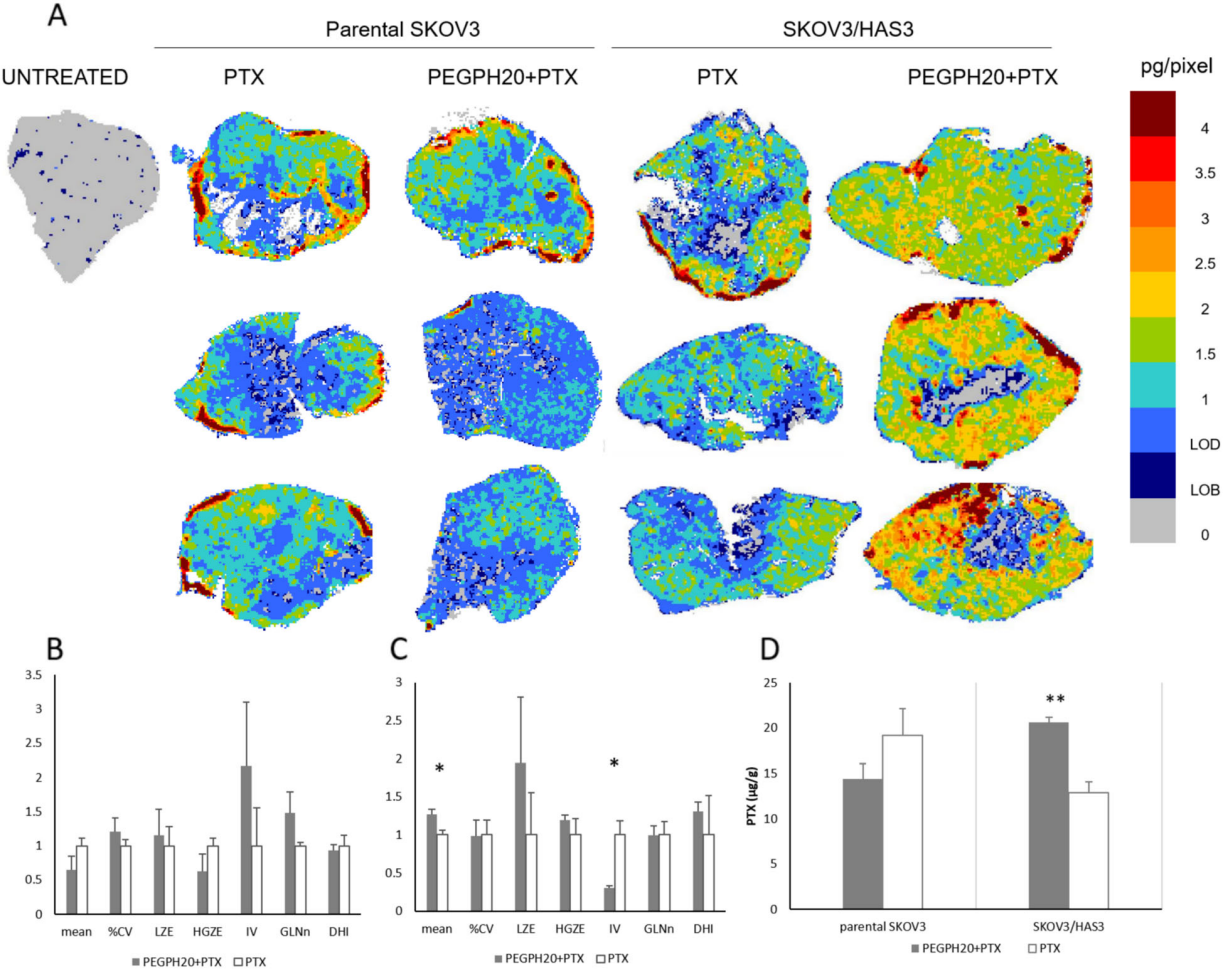
**A** Mass spectrometry images of PTX distribution in tumour tissues of parental SKOV3 or SKOV3/HAS3 after repeated treatment, q7dx2. Three tumours were analysed for each group. One representative section of three analysed for each tumour is shown. **B** GLSZM features (mean, CV%, LZE-Large-Zone Emphasis, HGZE-High Grey-level Zone Emphasis, IV-Intensity Variability, GLNn-normalised Grey-Level Non-uniformity and DHI-Drug Homogeneity Index) describing PTX distribution in SKOV3 or **C** SKOV3/HAS3 tumours 4 h after PTX, with or without PEGPH20 pre-treatment (repeated treatment, q7dx2) * Student's t test: *p*-value< 0.05. **D** Tumour concentrations of PTX in the second half of the same tumours analysed for MSI after PEGPH20 or vehicle pre-treatment (repeated treatment, q7dx2) **Student's t test: *p*-value< 0.01

MSI analysis was done following the same therapeutic regimen as PEGPH20 and PTX used for antitumor activity experiments (weekly PTX alone or after PEGPH20 0.1 mg/kg, 24 h before each dose of PTX). The results confirmed that PEGPH20 pre-treatment improved drug distribution in SKOV3/HAS3 but there were no differences between the two treatments in the parental SKOV3 model (Fig. 4A). The complete panel of GLSZM features is shown in Additional file 1. The GLSZM features analysis confirmed the pattern of alteration induced by PEGPH20 in the SKOV3/HAS3 model consistent with an easier diffusion of the drug (especially the statistically significant decrease of the IV, p-value 0.019). Moreover, the increase in drug concentration at the pixel level (“mean” features) measured by MSI became statistically significant (p-value = 0.039) after the second PEGPH20 and PTX doses, pointing out once again the better drug penetration (Panel 4B, C). This is confirmed by the increased total drug concentration measured by HPLC in these tumours 4 h after the last PTX dose after PEGPH20 pre-treatment only in SKOV3/HAS3 tumours (Fig. 4D). These results supported the hypothesis that HA depletion improves tumour penetration of drugs.

### PEGPH20 influences PTX distribution in the BxPC3 xenograft tumour model, modifies tumour tissue architecture and improves antitumor activity

**Fig. 5:**
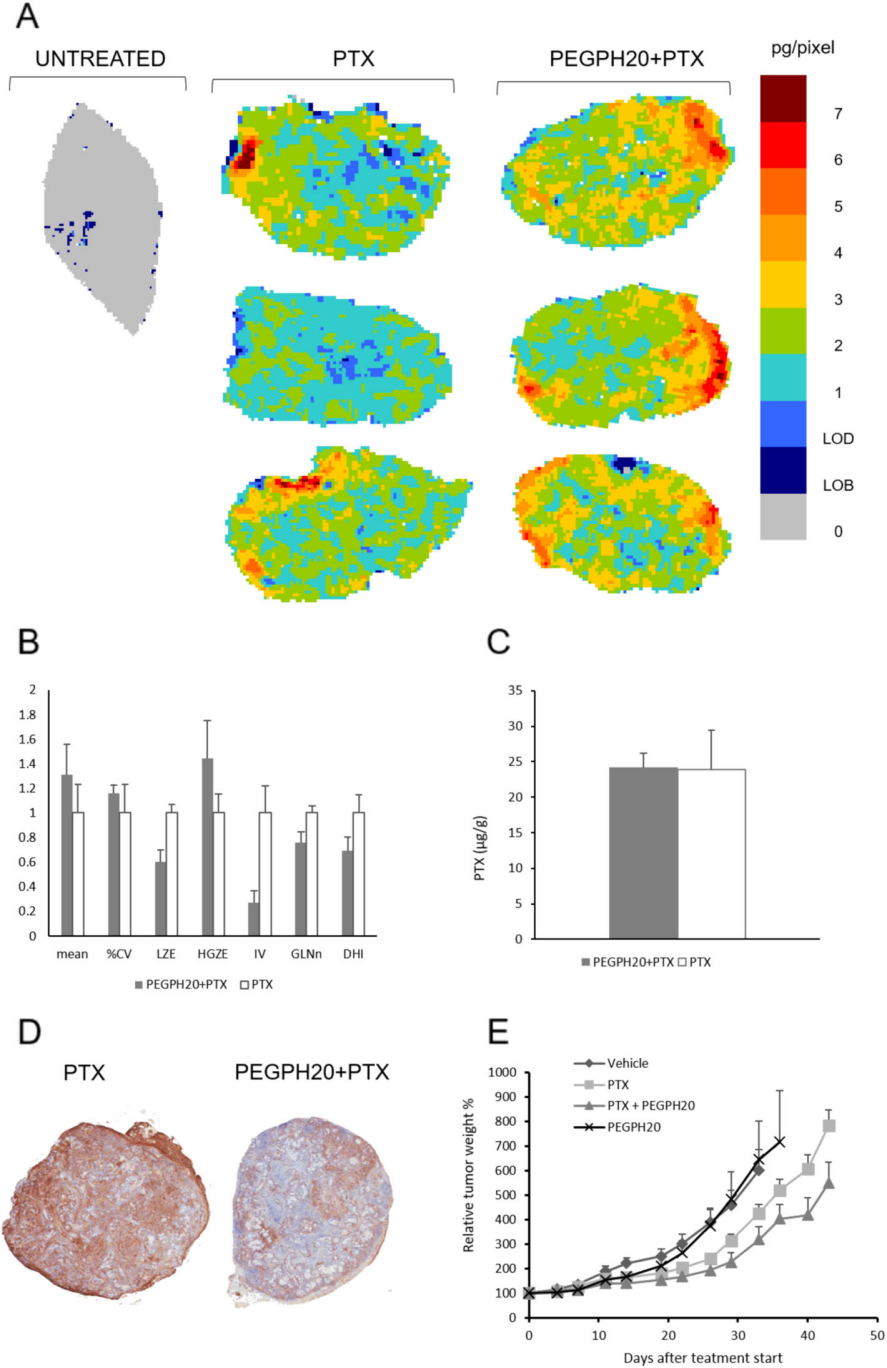
**A** Mass spectrometry images of PTX distribution in BxPC3 tumour tissues after PEGPH20 or vehicle pre-treatment; three tumours were analysed for each group. One representative section of three analysed for each tumour is shown; **B** The features describing drug distribution (mean, CV%, LZE-Large-Zone Emphasis, HGZE-High Grey-level Zone Emphasis, IV-Intensity Variability, GLNn-normalised Grey-Level Non-uniformity and DHI-Drug Homogeneity Index) and **C** tumour concentrations of PTX 4 h after the last treatment. **D** Representative HA staining images in the BxPC3 tumour, in PTX and PTX + PEGPH20 treated animals. **E** Tumour growth of BxPC3 bearing mice (*n* = 8) after treatment with PEGPH20 and PTX singly or in combination

The clinical relevance of our results was verified by testing the effect of PEGPH20 on PTX distribution in a xenograft model of pancreatic cancer, a tumour known for its dense, desmoplastic stroma with abundant ECM. The MSI analysis of PTX results are shown in Fig. 5. PTX alone, penetrated the tissue of BxPC3 tumours quite efficiently; however, the distribution of PTX was improved further after PEGPH20 pre-treatment (Fig. 5A).

Quantitative analysis of the MSI images indicated that PEGPH20 in BxPC3 induces a pattern of modifications in our panel of features similar to that of the SKOV3/HAS3 model: an increase of the mean pixel drug concentration, with lower LZE, higher HGZE and lower IV (Fig. 5B). These alterations described an overall picture where wider areas with low drug concentrations characterize the tumours from mice treated with PTX alone, while more high concentration zones were detected in tumours from mice pre-treated with PEGPH20, indicating easier drug penetration. The complete panel of GLSZM features is shown in Additional file 1.

No difference was seen between the experimental groups in PTX total concentrations measured by HPLC in tumour homogenates (Fig. 5C). The BxPC3 tumour had a high HA content and a significant reduction in HA specific staining after PEGPH20 treatment (PEGPH20 pre-treatment plus PTX 45.5 ± 7.6% HA positive pixels vs PTX alone 69.4 ± 7.8%, *p*-value 0.019; Fig. 5D). This is in line with the antitumor activity results. As reported in Fig. 5E and Additional file 1, PEGPH20 was completely inactive. PTX alone reduced the tumour growth rate, leading to a best T/C of 61.7 on day 26. The combination gave a modest, not significant, improvement of treatment activity with best T/C of 49.6 on day 29.

## Discussion

Heterogeneous spatial distribution of drugs in solid tumours represents a sort of pseudo-chemoresistance, because tumour cells can survive therapy simply because of lack of exposure [44]. This is partly due to the altered structure of the TME that acts as an obstacle to the drug penetration. Although intra-tumour pharmacokinetics is seldom taken into consideration, this could be an important point to act on to improve therapeutic efficacy.

HA is a TME component that is often overexpressed in various tumours [21, 22]. HA concentrations in clinical ovarian cancer biopsies range from 1 to 1000 ng/mg proteins and strongly correlate with tumour grade [45]. HA levels have been identified as predictive marker in ovarian cancer, with high levels indicating worse prognosis and lack of response to therapies [46, 47].

PEGPH20 was developed for its ability to disrupt HA in tumour tissue, inhibiting HA’s protumor properties, reducing TIFP and solid stress. Indeed, PEGPH20 can be exploited in a combination strategy, to improve the penetration of concomitant drugs through tumour tissue. Previous preclinical studies have shown that PEGPH20 increases the delivery of doxorubicin in pancreatic cancer [28] and favours tumour uptake of liposomal doxorubicin in prostate carcinoma [21, 24, 48]. In a xenograft model of ovarian cancer, HA depletion by PEGPH20 enhanced trastuzumab access to the tumour bulk [26].

In our study, we selected a HA-overexpressing ovarian cancer cell line and its parental counterpart as tumour models with different HA contents. Cells were implanted ectopically in nude mice. PEGPH20 pre-treatment increased PTX antitumor activity in the ovarian cancer model with high HA levels. In the parental model expressing lower HA levels, PEPGH20 did not affect PTX efficacy. Consistently with clinical data, we also found that the PTX monotherapy was less efficacious in ovarian cancer with high HA levels than in the parental cell line, supporting the correlation between HA content and tumour aggressiveness, chemoresistance and worse prognosis [31].

A single pre-treatment with PEGPH20 did not significantly increase PTX levels in the tumour, although there was a positive tendency. Plasma and liver levels of PTX were also similar, or even lower, in PEGPH20-pretreated mice than control ones. These pharmacokinetic results were really consistent with the reported data on effect of PEGPH20 pre-treatment on systemic and tissue distribution PEGylated polystyrene nanoparticles for drug delivery in HA expressing tumours [49]. Instead, the PTX concentrations in SKOV3/HAS3 tumours were clearly higher after two doses of the combination PEGPH20 plus PTX (Figs. 3 and 5). These data support the hypothesis that a single dose of PEGPH20 may not be enough to raise the total PTX levels significantly in a tumour mass in the SKOV3/HAS3 model (Figs. 2 and 3), and two doses of PEGPH20 are required to fully remodel the TME, increasing the tumour uptake of PTX. However, after a single dose of PEGPH20, TME remodelling is already under way, as suggested by IHC and MSI results. This technology gives direct visualization of PTX distribution in tumour tissue with the high sensitivity and specificity typical of mass spectrometry and good spatial resolution [37, 38]. This technology combined with an ad hoc protocol of image analysis clearly showed that PEGPH20 pre-treatment enhanced PTX distribution in the tumour tissue. SKOV3/HAS3 tumours had scarce PTX distribution, with some areas where the drug was undetectable. This was paralleled by modifications of tissue morphology by PEGPH20, particularly a striking reduction of interstitial space and a reduction in HA content (Fig. 4).

The effect of PEGPH20 on tumour PTX distribution and antitumor activity was at least partly confirmed in the BxPC3 xenograft, even though the results were less striking. Also in this model, PEGPH20 reduced HA tumour content and slightly improved PTX distribution and antitumor efficacy.

The data obtained in the pancreatic model may help to interpret the contradictory results of clinical trials. A phase II trial in patients with HA-high metastatic PDA (HALO 202) [33] showed significant improvement in PFS. The concomitant phase Ib/II trial (SWOG 1313) [50] instead showed a detrimental effect of PEGPH20 addition to FOLFIRINOX treatment in metastatic PDA patients. Unfortunately, in a phase III trial, PEGPH20 did not increase survival in patients with high-HA PDA compared to standard therapy (HALO 109–301) [34]. This failure suggests that targeting tumour stroma alone in pancreatic cancer is not enough to overcome chemoresistance since additional intrinsic factors surely have important roles [51].

Nonetheless, on the basis of our preclinical data, further clinical development of PEGPH20 should not be excluded, as certain tumours (e.g. ovarian cancer) and therapeutic settings could benefit. Further studies are still required to understand the interplay between TME, tumour cells and drug distribution better.

## Conclusions

This study demonstrated that PEGPH20 pre-treatment can improve PTX distribution in tumour tissue in two models with high HA levels, and ultimately increase PTX antitumor efficacy. Thus remodelling the extracellular matrix of HA-rich tumours is a promising new strategy to improve the intratumor distribution of anticancer drugs, increasing their therapeutic efficacy, without increasing toxicity.

## Supplementary Information

**Additional file 1.** Additional methods and results sections.

## Data Availability

The datasets generated and/or analysed during the current study are available from the corresponding author on reasonable request.

## Abbreviations

DHI: Drug homogeneity index
ECM: Extra Cellular Matrix
GLSZM: grey-level size-zone matrix
HA: Hyaluronic Acid
HAS: Hyaluronic Acid Synthase
HGZE: High Grey-level Zone Emphasis
HPLC: High Performance Liquid Chromatography
IV: Intensity Variability
LZE: Large Zone Emphasis
MALDI: Matrix Assisted Laser Desorption Ionization
MSI: Mass Spectrometry Imaging
PDA: Pancreatic Ductal Adenocarcinoma
PEGPH20: PEGylated recombinant human hyaluronidase PH20
PTX: Paclitaxel
RTW: Relative Tumour Weight
TIFP: Tumour interstitial fluid pressure
TME: Tumor Microenvironment
TW: Tumor weight
