# Supplementary material for: PEGylated recombinant human hyaluronidase (PEGPH20) pre-treatment improves intra-tumour distribution and efficacy of paclitaxel in preclinical models
Source: J Exp Clin Cancer Res. 2021 Sep 10;40:286. doi: 10.1186/s13046-021-02070-x (PMC8434701; doi:10.1186/s13046-021-02070-x)
Supplement: Supplementary file 1 — Additional file 1. Additional methods and results sections. [file 13046_2021_2070_MOESM1_ESM.docx]

**Title:** PEGylated recombinant human hyaluronidase (PEGPH20) pre-treatment improves intra-tumour distribution and efficacy of paclitaxel in preclinical models. ~~A new therapeutic strategy.~~

**ADDITIONAL FILE**

**Authors:**

Lavinia Morosi^1^*, Marina Meroni^1^*, Paolo Ubezio^1^, Ilaria Fuso Nerini ^1^, Lucia Minoli ^2,3^, Luca Porcu^1^, Nicolò Panini^1^, Marika Colombo^1^ Barbara Blouw^4^, David W.Kang^5^, Enrico Davoli ^6^, Massimo Zucchetti^1^, Maurizio D’Incalci^1§^ and Roberta Frapolli^1§^

^1^ Istituto di Ricerche Farmacologiche Mario Negri IRCCS, Department of Oncology

^2^ Department of Veterinary Medicine, University of Milan, Lodi, Italy

^3^ Mouse and Animal Pathology Laboratory (MAPLab), Fondazione UniMi, Milan, Italy

^4^ Biocept, San Diego, California

^5^ Halozyme Therapeutics, San Diego, California

^6^ Istituto di Ricerche Farmacologiche Mario Negri IRCCS, Laboratory of Mass Spectrometry

* L.M. and M.M. contributed equally

^§^ M.D. and R.F. co-last authors

**Corresponding author:**

Roberta Frapolli, ORCID ID 0000-0003-2907-273X

Istituto di Ricerche Farmacologiche Mario Negri IRCCS, via M. Negri 2, 20156 Milano

Phone: +39 02 39014233

Fax: +39 02 39014734

Email: [roberta.frapolli@marionegri.it](mailto:roberta.frapolli@marionegri.it)

L.M. and I.F.N. present address: IRCCS Humanitas Research Hospital, Via Manzoni 56, 20089 Rozzano – Milan, Italy

M.D. present address: IRCCS Humanitas Research Hospital, Via Manzoni 56, 20089 Rozzano – Milan, Italy; Department of Biomedical Sciences, Humanitas University, Via Rita Levi Montalcini 4, 20090 Pieve Emanuele – Milan, Italy

**Supporting material and methods**

*In vitro cytotoxicity*

A clonogenic assay was performed by plating the SKOV3 and SKOV3-HAS3 cell lines in six-well plates at the concentration of 3000 cells/well. 72 h after seeding, the cells were treated with different concentrations of paclitaxel (from 50 to 1000nM) for 24 h. 10 days after the seeding, colonies were stained and fixed with crystal violet solution containing ethanol and counted using a plate reader.

*Antitumor activity*

For analysis of the tumour growth curves, each tumor weight (TW) was normalized to the tumour weight of the same mouse at the start of treatment. The efficacy of the treatment was evaluated from the normalized TW curves of individual mice using two independent parameters: tumour growth (Gr, often referred to as “growth inhibition” during treatment) and the absolute growth delay (AGD)^1^. The percentage of Gr is indicative of the short-term antiproliferative effect. It measures the relative tumour growth between the start (day 0) and the end (day X) of treatment and was calculated adapting the NCI definition^2^ to the case:

%Gr_0-X_ = 100 x ΔTW*T_0-X_* **/** ΔTW*C_0-X_* when ΔTW*T_0-X_* ≥ 0 (TW increases)

%Gr_0-X_= 100 x ΔTW*T_0-X_* **/** <TW*C_0_*> when ΔTW*T_0-X_* < 0 (TW decreases),

where ΔTW*T_0-X_* = (TW*T_X_* - TW*T_0_* ) is the increment (decrement when negative) of the TW between day 0 and day X of a treated (*T*) tumour under analysis, ΔTW*C_0-X_* = (<TW*C_X_*> - <TW*C*_0_>) is the same increment as averages in the control (C) group (both TW*T_0_* and <TW*C_0_*> are equal to 1 by effect of the previous normalization). AGD, indicative of the long-term delay of tumor regrowth, was calculated as the difference (in days) between the time to reach a target size in a treated tumor and the mean time to reach the same size in the control group^3,4^. Depending on the different tumor growth curves of the two SKOV3 models and the BxPC3 model, AGD was calculated at 8, 6 and 4 times the size at the start of treatment (AGD8, AGD6 and AGD4, respectively).

*Pharmacokinetic analysis*

Mice bearing SKOV3 or SKOV3/HAS3 tumors of approximately 200-500 mg were treated with PTX (20 mg/kg, single dose), alone or after PEGPH20 (0.1 mg/kg, 24h before PTX). Biological samples (plasma, tumour, liver) were collected 1, 3, 8, 24 and 48 h after PTX (3 mice/group). Mice were anesthetized with isoflurane, blood was collected from the retro-orbital plexus into heparinized tubes, centrifuged for 10 min at 4000g at 4°C and the collected plasma was frozen at -20°C. After euthanasia, tumour and liver were immediately frozen in dry ice. PTX concentrations were measured in all the biological specimens by HPLC or LC-MS/MS. Analytical methods are described in previous publications ^5,6^. Pharmacokinetic parameters were calculated using Pk Solver, an add-in program for Microsoft Excel^7^.

**Supporting results**

*In vitro cytotoxicity*

SKOV3-HAS3 were intrinsically more sensitive to PTX than the parental line, being the IC50 of PTX 200 nM and 300-400 nM in SKOV3-HAS3 and SKOV3 cell lines, respectively (Figure S1).


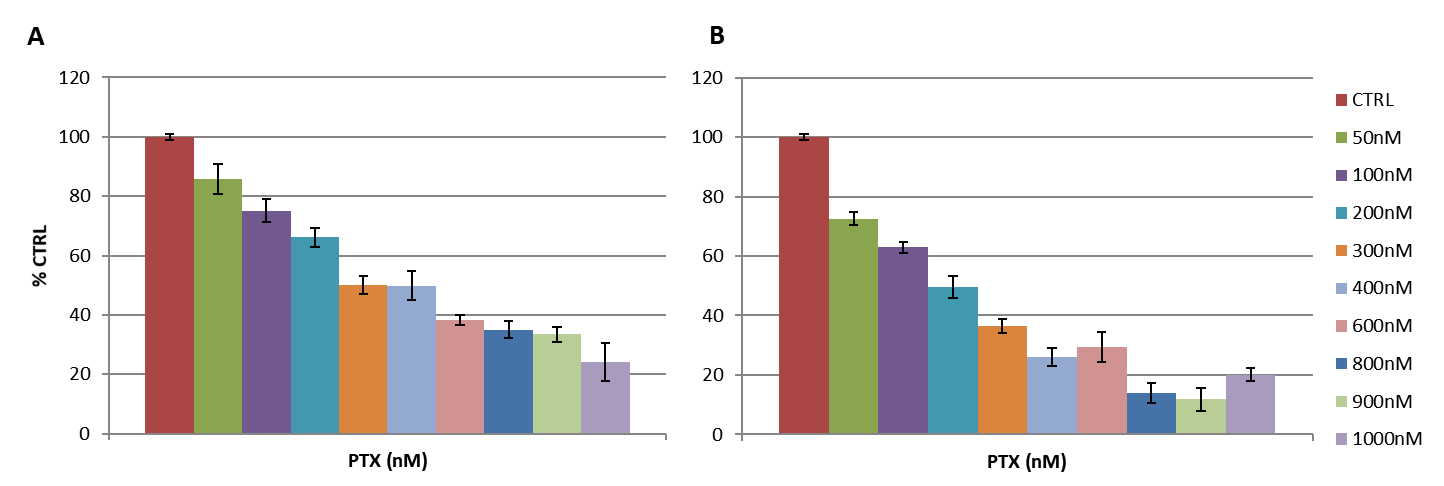


**Figure S1.** Inhibition of cell growth by PTX at different doses in SKOV3 (A) and SKOV3-HAS3 cells. The IC50 (dotted black line) is lower in SKOV3-HAS3 (200nM versus 300-400nm).

*Antitumor activity*

The analysis of the response to treatment has considered the short- and long-term characteristics of the tumour growth curves for each mouse. A score of the short-term response to treatment was obtained based on the growth inhibition measured on the increase/decrease of TW at the end of treatment compared to the volume at the start of treatment (%Gr_0-X_). The long-term score was the AGD calculated as the time to reach a target relative volume (4, 6 or 8 times the size at the start of treatment depending on the tumor model) minus the median time to reach the same target in the control group.

As shown in Figure S2 panels A, PTX induced a reduction of tumour growth (%Gr 59%, AGD6 =12 days) in SKOV3, while PEGPH20 was completely inactive (%Gr = 110%, AGD6=0 days). The combination with PEGPH20 did not affect the tumour initial growth inhibition induced by PTX (%Gr= 54%) and showed a tendency to recover more rapidly (AGD6 = 7 days). The behavior was different in SKOV3-HAS3 (Figure S2 panel B): although hyaluronidase was almost completely inactive, its combination with PTX dramatically affected tumor growth compared to PTX alone, leading to strong growth inhibition with %Gr of 19.5% vs 54%, respectively (p=0.017) and a longer AGD8 with a mean value of 18 vs 6 days (p=0.004). In BxPC3 model (figure S2 panel C) PTX alone caused a reduction in tumor growth at short time points (%Gr = 54%) but it is rapidly recovered, resulting in an AGD4 of only 3 days. With the combination, there was a slight, but not significant, improvement of both the short- and long-term responses, with a %Gr of 34% and an AGD4 of 9 days.

**
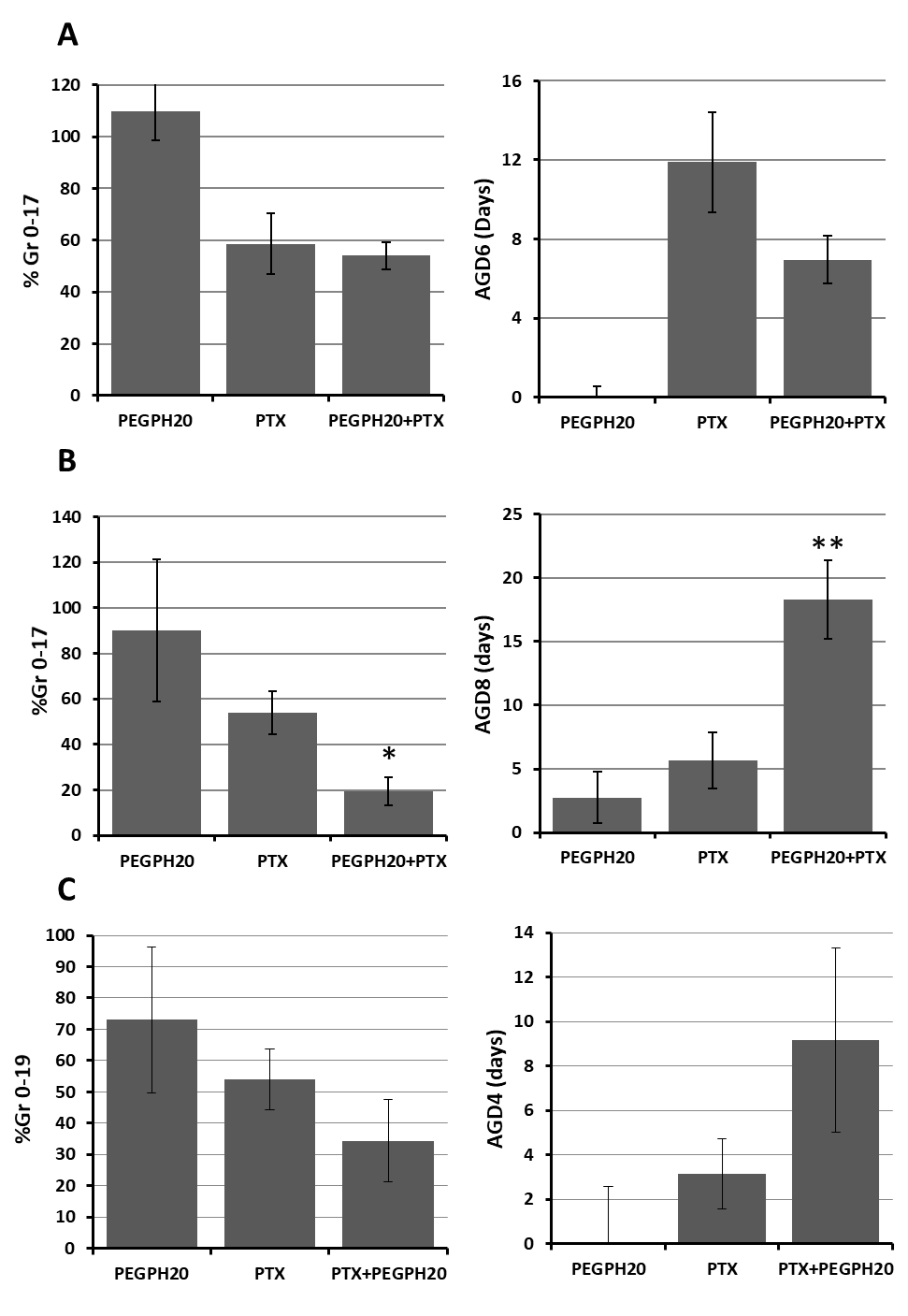
**

**Figure S2.** Parameters describing antitumor activity of PEGPH20 and PTX in (A) SKOV3, (B) SKOV3/HAS3 and (C) BxPC3 models (* p<0.05; **p<0.01).

*Pharmacokinetic analysis*

We measured PTX concentrations in tumour, plasma and liver at different times after a single dose of PTX 20 mg/kg, alone or after PEGPH20 pre-treatment. Results are depicted in Figure S3.

**
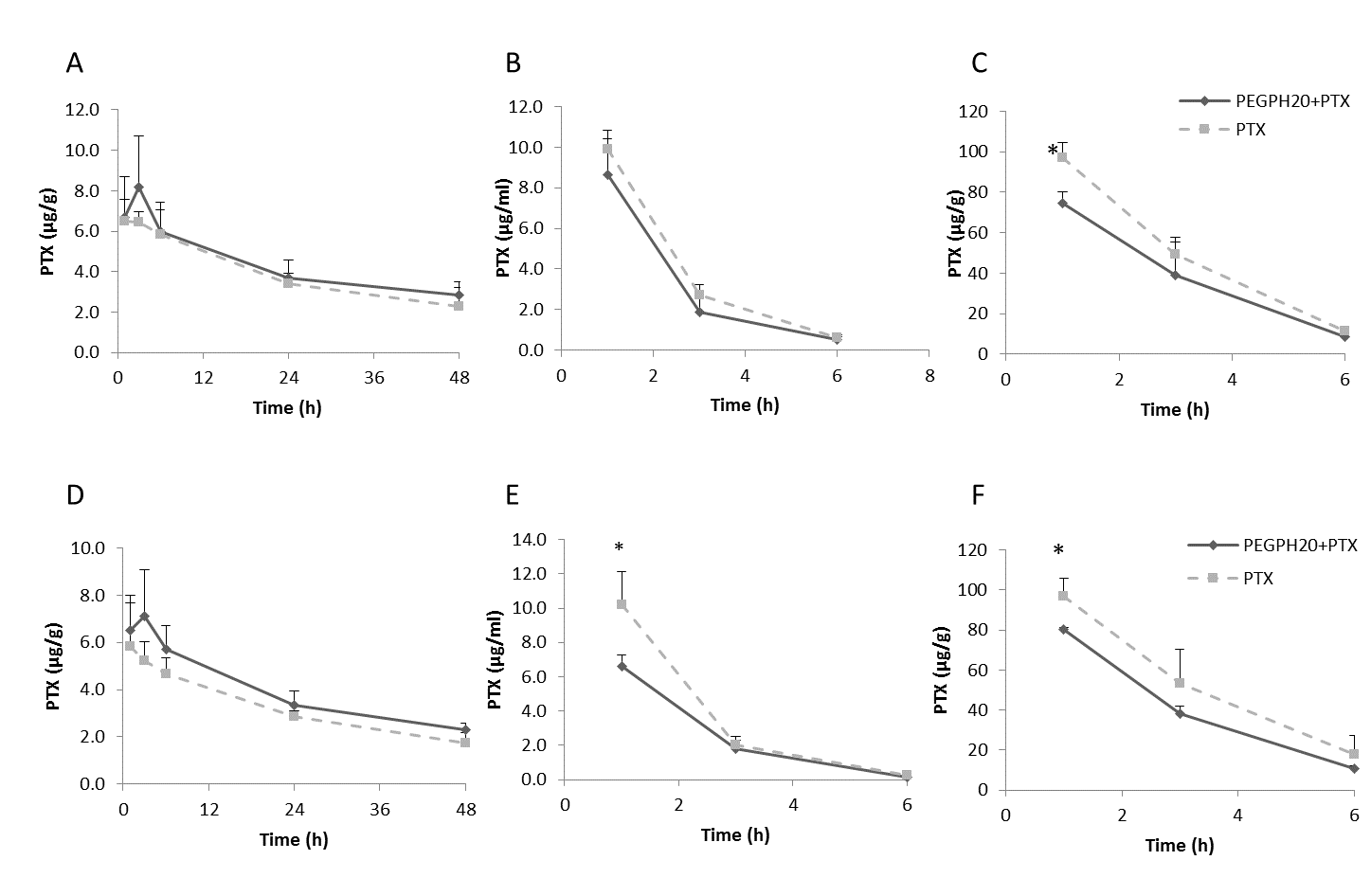
**

**Figure S3**. PTX concentrations in tumour (A, D), plasma (B, E) and liver (C, F) after a single dose of PTX 20 mg/kg, with or without PEGPH20 pre-treatment in the parental SKOV3 model (A-C) and SKOV3/HAS3 model (D-F) (* t test p value<0.05).

PTX concentrations in tumours were comparable in both tumour models. There was a small, not significant increase of PTX levels in HAS3-overexpressing tumours, that was maintained at all time points. Similar levels were also found for plasma in the SKOV3 model while circulating PTX was lower after PEGPH20 than vehicle pre-treatment in SKOV3/HAS3 model 1h after PTX (PTX=10.19±1.97 µg/ml; PEGPH20+PTX=6.62±0.65 µg/ml; t test p-value=0.031).

This observation is reflected by the calculated pharmacokinetic parameters (Table S1) that show lower plasma Cmax, AUC and consequently a higher distribution volume (Vd) of PTX administered after PEGPH20 pre-treatment. PTX levels in liver were also lower reduced the combination in both experimental models 1h after PTX. PTX concentrations in plasma and liver were beneath the lower limit of quantification (40 ng/sample for plasma and 80 ng/sample for liver) in samples collected 24 and 48h after treatment.

TABLE S1: Pharmacokinetic parameters

| **Parental SKOV3** | | **AUC 0-last** | **AUC inf** | **C max** | **C last** | **Clp** | **Vd** | **T/2** |
| --- | --- | --- | --- | --- | --- | --- | --- | --- |
|  |  | hr*μg/ml (or hr*μg/g) | | μg/ml (or μg/g) | | L/hr/kg | L/kg | hr |
| **PLASMA** | **PTX** | 31.97 | 33.05 | 9.9 | 0.60 | 0.61 | 1.09 | 1.25 |
|  | **PEGPH20+ PTX** | 27.65 | 28.57 | 8.63 | 0.51 | 0.70 | 1.26 | 1.25 |
| **TUMOUR** | **PTX** | 186.55 | 291.51 | 6.49 | 2.30 |  |  | 31.63 |
|  | **PEGPH20+ PTX** | 204.69 | 373.01 | 8.20 | 2.87 |  |  | 40.65 |
| **LIVER** | **PTX** | 286.37 | 312.09 | 97.25 | 11.23 |  |  | 1.59 |
|  | **PEGPH20+ PTX** | 222.53 | 241.45 | 74.76 | 8.39 |  |  | 1.56 |
| **SKOV3/HAS3** | | **AUC 0-last** | **AUC inf** | **C max** | **C last** | **Clp** | **Vd** | **T/2** |
|  |  | hr*μg/ml (or hr*μg/g) | | μg/ml (or μg/g) | | L/hr/kg | L/kg | hr |
| **PLASMA** | **PTX** | 32.19 | 32.58 | 10.19 | 0.28 | 0.61 | 0.86 | 0.97 |
|  | **PEGPH20+ PTX** | 20.97 | 21.14 | 6.62 | 0.13 | 0.95 | 1.19 | 0.87 |
| **TUMOR** | **PTX** | 150.97 | 224.07 | 5.84 | 1.72 |  |  | 29.46 |
|  | **PEGPH20+ PTX** | 185.64 | 291.06 | 7.11 | 2.28 |  |  | 32.05 |
| **LIVER** | **PTX** | 305.12 | 357.92 | 96.98 | 17.93 |  |  | 2.04 |
|  | **PEGPH20+ PTX** | 231.55 | 258.43 | 80.12 | 10.81 |  |  | 1.72 |

*GLSZM features*

The complete panel of GLSZM features proposed to describe tumour drug distribution, visualized by MSI are presented in Figure S3. The drug concentration at the pixel level was higher in the PEGPH20+PTX group (“mean” feature) with a lower %CV, suggesting a facilitated drug diffusion. The other features were based on the grey-level size-zone matrix, dividing the image into zones where the drug concentrations were in the same range (same grey level). Zone percentage (ZP) is the percentage of zones over the number of pixels (i.e. the size) of the image: lower values indicate that the image is made of a few large zones with similar drug concentration and higher values indicate greater fragmentation of the image in small zones. In SKOV3/HAS3 ZP was not affected by PEGPH20 pre-treatment.

Large-Zone Emphasis (LZE) is associated with the presence of wide areas with similar drug concentrations, either low or high, and High Grey-level Zone Emphasis (HGZE) indicates the presence of areas with high drug concentrations, regardless of whether the areas are small or large. Large-Zone High Grey-level zone Emphasis (LZHGZE) focuses on the wide areas at high drug concentration. Intensity Variability (IV) is highest when there are few large zones with low drug concentration. It decreases when the concentration increases (reaching higher levels) and the few large zones of low concentrations are fragmented. In the dynamics of drug arrival and diffusion, we expect that low drug concentrations are initially reached everywhere and then drug concentrations gradually rose starting from smaller areas where physical barriers are weaker. In SKOV3/HAS3 PEGPH20+PTX showed a tendency to fewer large zones (lower LZE than PTX treatment) and more higher drug concentrations zones (higher HGZE). Because High-drug-concentration zones are not wide, their presence is less evidenced by LZHGE (only slightly higher in PEGPH20+PTX than PTX alone treatment). The PEGPH20 effect is particularly reflected by a reduction of intensity variability (IV), consistent with higher diffusion of the drug, reaching higher concentrations.

Other features considered were: the normalised Grey-Level Non-uniformity (GLNn), highest when zones concentrate at a single grey level, but poorly sensitive to variations when more grey levels are involved; the average size of the zones (ZSµ) was expected to increase homogeneous concentrations in larger zones (regardless of the grey level) but was strongly affected by the large number of small zones; and the Drug Homogeneity Index (DHI), a recently proposed feature measuring the average area of the zones with larger sizes (over a given arbitrary threshold) as a fraction of the whole image area. Neither of these features was appreciably affected by PEGPH20 pre-treatment, although different thresholds were considered for calculating DHI (Figure S3). In parental SKOV3 model, the pattern of alterations of imaging features detected in the SKOV3/HAS3 model was not seen. Features calculated in tumours treated with PEGPH20+PTX were almost undistinguishable from those in tumours treated with PTX alone. Consistent results were obtained by GLSZM features analysis in parental SKOV3 and SKOV3/HAS3 after repeated treatments (Figure S4).

The results here are influenced by another variable: the amount of necrosis. Histological analysis of adjacent tissue slices revealed vast areas of necrosis in these tumour samples, probably as a consequence of the high TW and activity of PTX on tumour cells. We can speculate that the presence of necrotic areas impairs PTX distribution , as previously demonstrated^8^ and affects data interpretation. The LZE increase trend and LZHGE decrease trend in SKOV3/HAS3 can be due to this effect as well as the IV increase trend in parental SKOV3.

In the BxPC3 model, PEGPH20 induced a pattern of modifications of imaging features similar to that observed in SKOW3/HAS3 cells: an increase of the mean pixel drug concentration, with lower LZE, higher HGZE and lower IV. A joint interpretation of these modifications indicates that at the time when samples are taken (4h after drug administration) tumours treated with PTX alone have wider areas with low drug concentrations, close to our detection limit, while in PEGPH20+PTX treated samples drug reaches higher concentrations in some areas, indicating easier penetration. Consistently with this picture, in BxPC3 we also observed a tendency to lower GLNn and DHI values in PEGPH20+PTX than PTX alone treatment. Lower GLNn in PEGPH20 pretreated samples was due to a wider distribution of the zones through the grey levels, also in zones with high grey-levels ( i.e. higher drug concentration), while in PTX alone treated samples, the zones were more concentrated in the medium/low grey-levels. Lower DHI in PEGPH20 pre-treated samples was due to the presence of more zones above the size threshold, although with a lower average size than tumours treated with PTX alone.

Detection of the effects of PEGPH20 pre-treatment on GLNn and DHI features was facilitated in BxPC3 by a relatively high average drug concentration. The same effects could not be detected in SKOV3/HAS3 where drug concentrations were generally lower and closer to the detection limit.

*
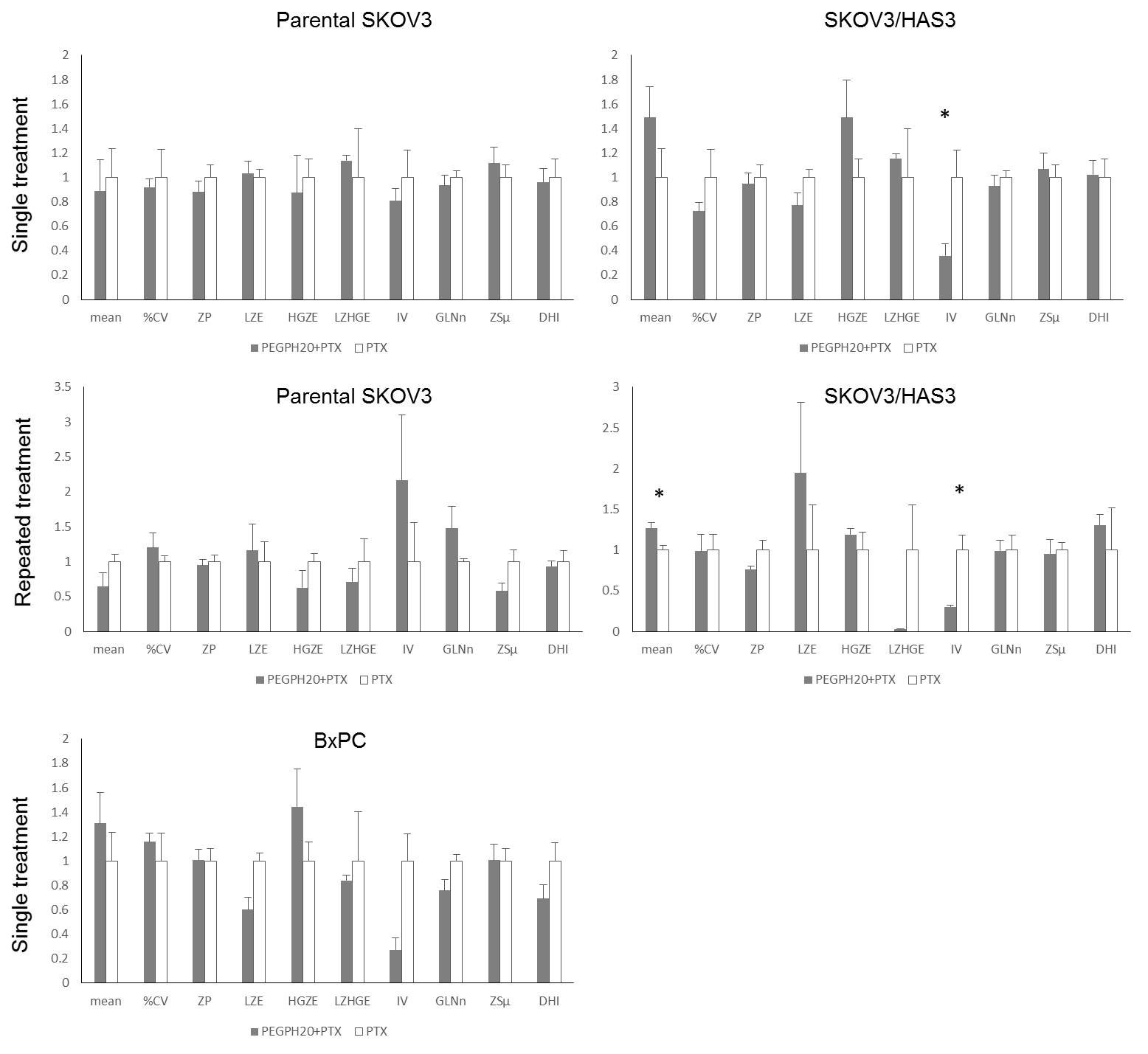
*

**Figure S4. GLSZM features.** Features describing drug distribution and influenced by PEGPH20 pre-treatment. The mean value of each feature was rescaled to the PTX mean for comparison (* p value<0.05).

*Histological analysis*

**Table S2** – Morphological analysis: parental SKOV3.

| **Treatment** | **Necrosis** | **Stroma** | **Loose arrangement of the tissue** |
| --- | --- | --- | --- |
| PTX | 5 | ++ | - |
| PTX | 0 | ++ | - |
| PTX | 50 | ++ | - |
| PTX | 0 | ++ | - |
| PEGPH20+PTX | 50 | ++ | - |
| PEGPH20+PTX | 10 | ++ | - |
| PEGPH20+PTX | 0 | ++ | - |
| PEGPH20+PTX | 0 | + | - |

**Table S3** – Morphological analysis: SKOV3/HAS3.

| **Treatment** | **Necrosis** | **Stroma** | **Loose arrangement of the tissue** |
| --- | --- | --- | --- |
| PTX | 50 | ++ | +++ |
| PTX | 10 | ++ | ++ |
| PTX | 15 | ++ | ++ |
| PEGPH20+PTX | 30 | ++ | - |
| PEGPH20+PTX | 5 | +++ | - |
| PEGPH20+PTX | 10 | ++ | + |

**Supporting References**

1. Ubezio, P. Beyond The T/C Ratio: Old And New Anticancer Activity Scores In Vivo. *Cancer Manag Res* **11**, 8529–8538 (2019).

2. Shoemaker, R. H. The NCI60 human tumour cell line anticancer drug screen. *Nat. Rev. Cancer* **6**, 813–823 (2006).

3. Vázquez, R. *et al.* Promising in vivo efficacy of the BET bromodomain inhibitor OTX015/MK-8628 in malignant pleural mesothelioma xenografts. *Int. J. Cancer* **140**, 197–207 (2017).

4. Bizzaro, F. *et al.* Tumor progression and metastatic dissemination in ovarian cancer after dose-dense or conventional paclitaxel and cisplatin plus bevacizumab. *Int J Cancer* **143**, 2187–2199 (2018).

5. Colombo, C. *et al.* PEGylated Nanoparticles Obtained through Emulsion Polymerization as Paclitaxel Carriers. *Mol. Pharm.* **13**, 40–46 (2016).

6. Fruscio, R. *et al.* Clindamycin-paclitaxel pharmacokinetic interaction in ovarian cancer patients. *Cancer Chemother. Pharmacol.* **58**, 319–325 (2006).

7. Zhang, Y., Huo, M., Zhou, J. & Xie, S. PKSolver: An add-in program for pharmacokinetic and pharmacodynamic data analysis in Microsoft Excel. *Comput Methods Programs Biomed* **99**, 306–314 (2010).

8. Giordano, S. *et al.* Heterogeneity of paclitaxel distribution in different tumor models assessed by MALDI mass spectrometry imaging. *Scientific Reports* **6**, (2016).
